# Climatic Droplet Keratopathy in Argentina: Involvement of Environmental Agents in Its Genesis Which Would Open the Prospect for New Therapeutic Interventions

**DOI:** 10.1155/2015/527835

**Published:** 2015-09-16

**Authors:** María Fernanda Suárez, Leandro Correa, Nicolás Crim, Evangelina Espósito, Rodolfo Monti, Julio Alberto Urrets-Zavalía, Horacio Marcelo Serra

**Affiliations:** ^1^CIBICI-CONICET, Facultad de Ciencias Químicas, Universidad Nacional de Córdoba, Haya de la Torre esquina Medina Allende, 5000 Córdoba, Argentina; ^2^Departamento de Oftalmología, Clinica Universitaria Reina Fabiola, Universidad Católica de Córdoba, Oncativo 1248, 5000 Córdoba, Argentina

## Abstract

Climatic droplet keratopathy (CDK) is a degenerative corneal disease of unknown etiology. We described CDK for the first time in Latin America in the Argentinean Patagonia (El Cuy). A deeper knowledge of CDK pathogenic mechanisms will provide new therapeutic strategies. For that reason we investigated the prevalence of CDK in El Cuy and its existence in other 3 provinces with similar climate. Patients eyes were examined, habits throughout lives were inquired about, and serum ascorbate (sAA) was determined. All individuals work outdoors for most of the day. All regions had normal O_3_ levels. Individuals from regions 1, 2, and 3 had very low consumption of vegetables/fruits and low sAA levels. Conversely, region 4 individuals had balanced diet and higher sAA concentrations. CDK was only found in region 3 where individuals had partial deficiency of sAA and did not use eye protection. No CDK was found in regions 1 and 2 where individuals had similar work activities and dietary habits to those in region 3 but wear eye protection. No disease was found in region 4 where individuals work outdoors, have balanced diet, and use eye protection. To summarize, the CDK existence was related not only to climate but also to the dietary habits and lack of protection from sunlight.

## 1. Introduction

In 1898, Baquis [1] described for the first time an acquired degenerative disease of the human cornea (colloid degeneration of the cornea), potentially disabling and being characterized by a slow progression to corneal opacity. The general clinical features of this disease, which occurs in individuals who inhabit certain arid regions of the world [2–5], were reported by Klintworth [6]. The first descriptions of the disease were given by Baquis in 1898 [1] and also in 1935 by Lugli [7]. In 1937, Zanettin found climatic droplet keratopathy (CDK) in fishermen from the Dahlak Archipelago of the Red Sea and described a severe form which leads to blindness [8]. Since then, the disease has been described in different parts of the world [9–33] and more recently in Argentina [34]. The severity of this disease can be classified in three stages, according to the portion of cornea involved and the clinical aspects:

- Grade 1: it is characterized by multiple small translucent subepithelial deposits, located near the temporal and/or nasal limbus, best seen with backscattered slit-illumination and high magnification. A prelimbal fringe of clear cornea is also frequently observed. At this stage, the visual acuity is not compromised.
- Grade 2: the opacity extends over the lower two-thirds of the cornea, giving a fuzzy appearance. The indemnity of the superior cornea, protected by the eyelid, suggests an etiologic factor contributing to chronic corneal exposure to ultraviolet radiation (UVR) and other stresses.
- Grade 3: it is characterized by the presence of clusters of golden subepithelial droplets of different sizes, about 1 mm diameter, which cover the cornea as the disease progresses. In advanced cases, areas of the anterior stroma with vascularization, opacification, or fibrosis may be seen. In general, the visual acuity is severely affected by this stage.

In addition to the findings related to the cornea, the solar radiation that chronically reaches the bottom and most exposed part of the iris could play an important role in inducing depigmentation or atrophy in superficial layers, as previously observed in some patients with CDK [35].

Although globular deposits in the anterior layer of the corneas with CDK were described many years ago using optical and electron microscopy [36, 37], more recently, these anomalies have been further characterized by us using* in vivo* confocal microscopy (IVCM) [38].

Although some components in the corneal droplets have been identified [39, 40], the exact composition still remains unknown. A few years ago, Fujii et al. prepared and characterized a polyclonal antibody against peptides containing D-*β*-Asp [41, 42], and the same authors later showed that CDK and pinguecula samples were immunoreactive for peptides containing D-*β*-Asp and advanced glycation end products (AGEs) [43, 44].

Tears contain many identifiable proteins [45], with the variation in their composition possibly defining biomarkers that could lead to a better understanding of the underlying pathology [46]. As CDK is an ocular surface disease and the analysis of the tear proteins may result in further understanding of this disease we studied tears glycoproteins in CDK patients using glycopeptide capture techniques and proteomics. Our results suggest that the enzymatic glycosylation may also be involved in the formation of deposits in CDK, since altered levels of N-glycosylation of certain proteins were observed in the tears of patients with CDK [47].

We have also investigated matrix metalloproteinases (MMP) and their inhibitors, TIMP, in patients with CDK, because these molecules control the degradation of the corneal epithelium and stroma. We showed increased levels of gelatinases and proinflammatory cytokines, as well as decreased expression of TIMP-1 in tears and biopsies of patients with CDK. Similar results were obtained when corneal epithelial cells were exposed to UVR* in vitro* [48, 49]. This data suggests that the pathogenesis of this disease is partly driven by a significant inflammatory response with the poor antiproteolysis shield making the cornea more vulnerable to increased levels of MMP.

The present study was conducted in order to investigate if nutrition, work activity, and eye protection from solar radiation are involved in the development of CDK. We carried out this research by studying individuals who inhabit a region of Argentinean Patagonia where we have found patients with this corneal disease (El Cuy department in the Argentinean Patagonia) and in other three Argentinean regions with climatic conditions similar to those of El Cuy department.

Even though this disease has received different denominations over the years, CDK being the most common name, we shall try to convince the scientific community that, based upon the results of this paper, a more accurate name for this corneal pathology would be environmental droplet keratopathy, rather than climatic droplet keratopathy.

## 2. Materials and Methods

### 2.1. Geographical and Climatic Characteristics of the Regions

**Figure 1:**
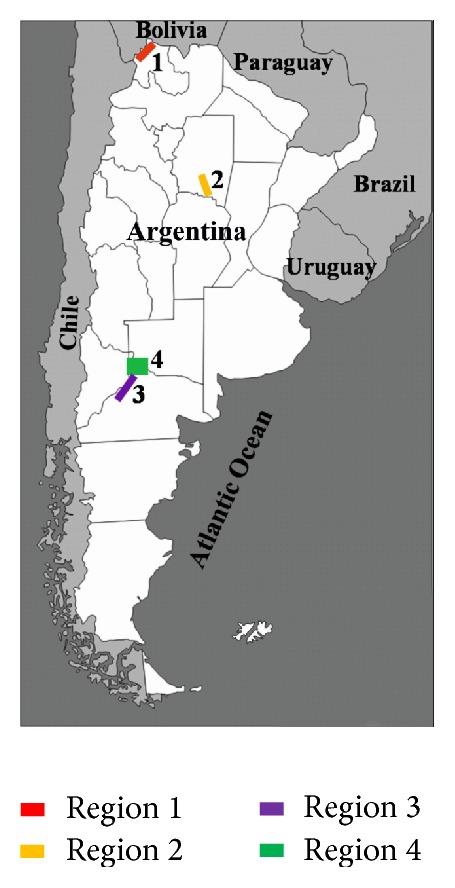
Map of the four regions included in this study.

Figure 1 shows the different regions studied.

*Region 1*. Santa Catalina department, in the province of Jujuy, is located in the Altiplano region of Puna, at an average height of 3700 meters above sea level, between 65°53′–66°45′ west longitude and 21°47′–22°15′ south latitude. It is located about 2250 km from the Atlantic Ocean with its northern limit being Bolivia. This region has a cold arid climate (Bwk; Köppen climate classification); see World Map of Köppen-Geiger climate classification direction insert: http://koeppen-geiger.vu-wien.ac.at/shifts.htm (accessed January 9, 2015).

The soil is clayey-sandy and covered by low shrub or steppe vegetation. It presents a high diurnal range of temperature, with very low annual rainfall (100–200 mm) and scarce clouds. The region suffers the constant action of strong winds as also found in Patagonia, with the average temperature being below 18°C.

*Region 2*. Quebrachos department, in the south of the province of Santiago del Estero, is located between 200 and 500 meters above sea level, between 63°13′–63°28′ west longitude and 28°59′–29°45′ south latitude. The region has a subtropical climate (Csa/Csb; Köppen climate classification) with an annual average temperature of 21.5°C and extreme variations of up to a maximum of 45°C. The rainfall is greater during the summer months, with a maximum of 500 mm and a minimum of 300 mm. The strongest winds occur in winter with a mean speed of approximately 75 km/h. The vegetation is typical of the Chaco/Santiagueño native vegetation with the presence of quebracho, carob, mistol, chañar, and so forth.

*Region 3*. El Cuy department is located in the center/west of the province of Río Negro, 750 meters above sea level, between 67°54′–69°04′ west longitude and 38°56′–40°25′ south latitude. It is located 280 km from the border with Chile and 300 km from the Atlantic Ocean. This region has cold semiarid climate (Bsk, Köppen climate classification) with annual rainfall being less than 190 mm and a very low relative humidity. The region experiences hot dry summers (often exceptionally hot) and cold winters, with great diurnal temperature variations.

*Region 4*. General Roca department is located in the north of the province of Río Negro. This region lies between 66°56′–68°00′ west longitude and 38°56′–39°07′ south latitude, 370 meters above sea level and 400 km from the Atlantic Ocean and 340 km from the border with Chile. This region has a cold semiarid climate (Bsk, Köppen climate classification), but due to the intervention of man and water provided by irrigation canals it is a green valley with numerous farms. It is protected from winds by the edges of the plateau, which act as walls.

### 2.2. Type of Study and Individuals

This investigation was approved by the Institutional Review Board of the Catholic University of Córdoba and the Institutional Research Ethics Committee of Health, Ministry of Health of the province of Córdoba, Argentina (recorded in the RePIS), and carried out in accordance with the principles of the Statement of Helsinki.

Nonprobabilistic consecutive patients older than twenty years, who live during their entire life in any of these regions of Argentina and who agreed to take part in the study after reading a summary of the research project and signed a written consent, were examined by specialists in ophthalmology. The study sample was composed of 89, 134, 125, and 113 individuals for regions 1, 2, 3, and 4, respectively.

### 2.3. Ophthalmologic Examination

All subjects received a thorough ophthalmologic examination that included detailed examination of the anterior eye segment using a slit lamp (Slit-Lamp 100/16, Carl Zeiss, Oberkochen, Germany; AO Slit Lamp 11665, American Optical Co., Buffalo, USA; Led Slit Lamp XL-1, Shin-nippon, Ohira Co., Niigata, Japan). Many individuals also received a test of visual acuity with a Snellen chart and Landolt rings or E for illiterate individuals, objective refraction determination using an autorefractometer (RK1, Canon Inc., Tokyo, Japan). Three representative cases of patients with different grades of CDK were also studied using* in vivo* confocal laser scanning microscopy (IVCM) as previously described [38].

### 2.4. Lifestyle

All patients completed a questionnaire related to diet, work activity, and the wear of eye protection (sunglasses, hats) during their entire life.

### 2.5. Ascorbate Serum Concentration

The levels of serum ascorbate (sAA) were studied in twenty randomly selected participants from each region (age and gender matched) by high performance liquid chromatography (HPLC) using an LC-18 column (25 cm high × 4.6 mm diameter with a particle size of 5 *μ*m) as previously described [50]. For region 3 only CDK patients were studied.

### 2.6. Levels of Ozone

The total O_3_ column concentration average in a period of 10 years for each of the regions was obtained using the values of ozone from the website of the National Aerospatial Agency (NASA, USA): Total Ozone Mapping Spectrometer (TOMS-NASA), http://ozoneaq.gsfc.nasa.gov/tools/ozonemap/. For each year, four measurements were obtained on March 21, June 21, September 21, and December 21 in order to determine the annual average. The total O_3_ column concentration was measured in Dobson units (DU), considering that normal values range between 230 and 300 DU.

### 2.7. Statistical Analysis

The data were analyzed using different statistical tests such as Chi-Square test and analysis of variance (ANOVA) followed by post hoc least significance difference. The level of statistical significance was set at *p* < 0.005.

## 3. Results

Table 1 presents the data for the average age, gender distribution, and prevalence of CDK in individuals living in the four Argentinean regions. As it can be seen CDK was only observed in 25 individuals (8 women and 17 men) living in region 3 (20% prevalence). Grade 1 was observed in 15 individuals (60%), grade 2 in 7 individuals (28%), and grade 3 in 3 patients (12%). No cases were found in individuals younger than 38 years old.

**Figure 2:**
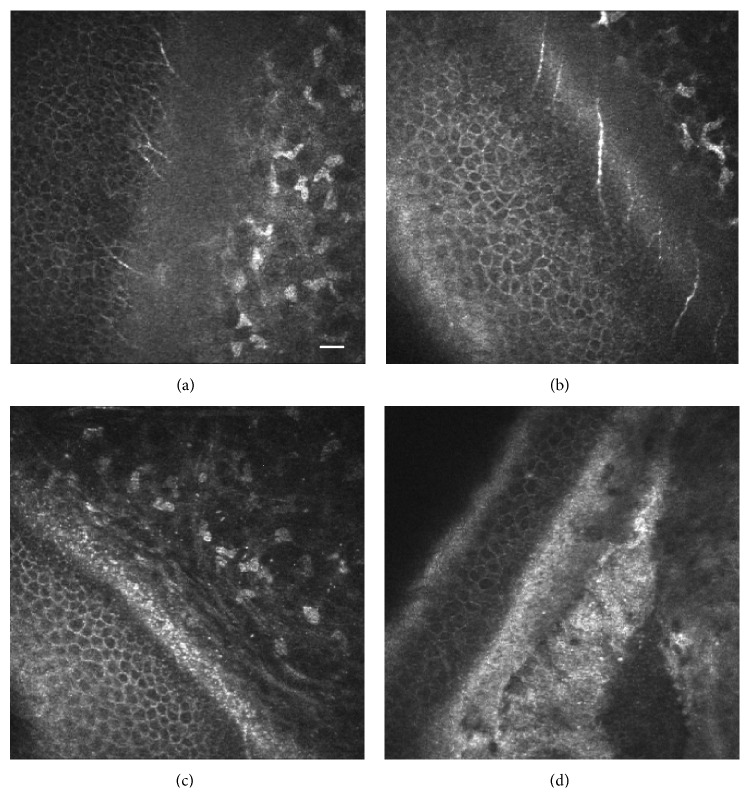
*In vivo* confocal microscopy showing oblique sections of the human cornea where the epithelium, Bowman's layer, and the anterior stroma can be observed. (a) Normal cornea without hyperreflective subepithelial deposits, (b) (grade 1) dot-like deposits at the level of Bowman's layer, (c) (level 2) showing an increase in the density of the hyperreflective dot-like deposits in Bowman layer and superficial stroma, and (d) (grade 3) hyperreflective and nonreflective condensation deposits in the Bowman layer and superficial stroma. Bar = 50 microns.

In Figure 2, IVCM oblique images of three representative patients with different grades of the disease clearly show the increase in hyperreflective dot-like deposits at the subepithelial layer as the disease progress.

Values of pterygium and pinguecula are summarized in Table 2. Pinguecula prevalence was significantly higher than pterygium for regions 1, 2, and 3, but not for region 4 (*p* < 0.0005). When we compared the prevalence of pterygium among the four regions there was no significant difference (*p* = 0.2457), whereas the prevalence of pinguecula was of significant difference (*p* < 0.0001).

In the same region where we found CDK there was the highest prevalence of the other two diseases. The pterygium and pinguecula prevalence were significantly higher in region 3, only when they were compared to values from region 4 (*p* = 0.0688 and *p* < 0.0001, resp.).

The principal labor activities for the different regions are summarized in Table 3. The occupation in region 1 is sheep and domestic camelid breeding, whereas for region 2 it is goat rearing, logging, and coal production. Individuals in region 3 are mainly dedicated to sheep farming, sheep shearing, and, in some cases, the manufacture of wool, while individuals in region 4 work on tasks related to the cultivation, harvesting, and packaging of pears, apples, and plums.

The main dietary habits of individuals in region 1 are the consumption of meat, quinoa, corn, and potatoes; in region 3 sheep meat is ingested two or three times a day, with small amounts of milk taken sporadically. Vegetables and fruit are exception items in the diet of the inhabitants of these regions. The diet of individuals in region 2 consists of some vegetables (potatoes, zucchini), some meat, and a scarce amount of fruits. In contrast, individuals inhabiting region 4 have a balanced diet (meat, vegetables, cereals, and fruit) (Table 3). In all regions, individuals manifest drinking yerba mate infusion (a local infusion).

Dietary deficiency in foods rich in AA was reflected in the low sAA levels found in the individuals living in regions 1, 2, and 3 (0.27 mg/dL ± 0.13, 0.31 mg/dL ± 0.11, and 0.21 mg/dL ± 0.09, resp.). These values differed significantly (*p* < 0.001) from individuals in region 4 (0.72 mg/dL ± 0.44).

As can also be observed in Table 3, more than 80% of individuals in region 1 use winged hats, whereas in region 2 sunglasses are worn. In region 3, most individuals do not wear glasses or hats, while in region 4 the majority of people studied use hats and/or sunglasses.

The twenty-five CDK patients manifested they worked outdoors in sheep farming, never used eye protection, had a very restricted diet as previously shown during their life, and had the lowest AAs concentration. As can be seen in Table 3 the majority of individuals from this region who do not suffer CDK have the same work activity and habits.

When the total O_3_ column concentrations (Dobson units) measured on March 21, June 21, September 21, and December 21 of each of the last ten years from each region were analyzed no significant differences at annual average were found (data not shown). We then added all the values corresponding to the last 10 years and obtained that the mean ± SD of total O_3_ column concentrations for regions 1, 2, 3, and 4 was 252.6 ± 14.5, 272.4 ± 16.7, 288.3 ± 10.2, and 273.7 ± 5.8, respectively. These results rule out any thinning of the ozone layer in the four studied regions.

## 4. Discussion

CDK has been defined as a rural disease in which the clinical presentation and severity of corneal injuries can vary significantly depending on the region and its weather. More severe forms of CDK have been described in arid regions with high temperatures, such as those of the islands of the Red Sea [8, 29], than in cold regions such as Labrador and the Arctic Circle [23].

Even though CDK is a well-differentiated clinical entity, with bilateralism being the rule and often asymmetry, a differential diagnosis with other entities is needed, such as secondary spheroidal degeneration, gelatinous drop-like dystrophy, corneal edema, band-shaped corneal degeneration, Salzmann's nodular degeneration, climatic stromal proteoglycan keratopathy, Vogt's limbal degeneration, superficial corneal dystrophies, and hypertrophic peripheral corneal degeneration. The combined use of slit lamp biomicroscopy and IVCM facilitate the diagnosis of this disease [35, 38].

Our hypothesis about the genesis of CDK is that in the cornea of those people chronically exposed to unfavorable environmental conditions (high exposure to UVR, lack of vegetation/shade, dry climate with windy conditions, airborne particle bombardment, partial AA nutritional deficiency, lack of eye protection, and genetic factors) an oxidative stress and inflammatory processes lead to a progressive degradation and accumulation of proteinaceous material in Bowman's membrane and the superficial stroma [51]. We have recently shown elsewhere that, in patients with CDK, a hypersensitive reaction occurred in the cornea with the initial participation of important proinflammatory components of the innate immune system [52]. We have previously shown a lack of correlation between genetic ancestry (as represented by haploid genetic systems) and the incidence of CDK in Argentina [53]. Also, in previous unpublished studies we have investigated the variation at the blood and HLA-DRB1 alleles groups between patients and relevant controls. Although we found that the expression of 0 Rh+ and DRB1∗14 was higher in CDK patients there were not any significant associations between these alleles and CDK. Since ALDH3A1 gene encodes a protein that protects the mammalian cornea from harmful UVR, we are currently studying genetic variation at this locus in the population that suffers from CDK.

Although the eye depends on the energy from visible radiation to carry out its fundamental physiological processes, it can also be damaged by this radiation as well as by UVR. Eye diseases in which sunlight is implicated are called ophthalmoheliosis, with these conditions representing important eye health hazards in many communities worldwide. However, the interpretation of clinical examination is complicated, because of the difficulty in measuring the quantity and exact wavelength of light to which an individual has been exposed, as well as the length of time over which an injury has been progressing [54].

Acute or chronic corneal exposure to UVR induces altered proteins, DNA fragmentation, free radical generation, lipid peroxidation, and so forth, resulting in actinic keratosis or keratopathies affecting primarily the epithelium and the anterior stroma. Other studies have shown that exposure to UVR is responsible for the development of cortical pterygium and cataracts [55].

The high content of AA, some proteins, and nucleic acids in the corneal epithelium function as a filter, by absorbing up to 77% of UVR of dangerous wavelengths [54, 56–58]. This vitamin is synthesized by different mechanisms both in the animal kingdom and in plants. However, bats, guinea pigs, and primate anthropoids, including humans, have lost the ability to synthesize AA as a result of mutations of L-gulono-*γ*-lactone oxidase (GLO) genes, and this powerful antioxidant must be incorporated in the diets of these animals (see [59]). Tears also have among different components that prevent oxidative stress a constant level of AA which is maintained by the lachrymal gland and not by the cornea [57, 60]. Our group, as others, working with guinea pigs fed on different doses of AA and exposed to UVR, have shown that cornea damage produced by radiation is related to the dose of AA consumed [59, 61, 62]. In humans, corneal haze after photorefractive keratectomy has only been reported in Norway, when the sun is visible 24 hours/day [63], but the administration of a dietary supplementation with AA during pre- and postoperative periods can reduce its incidence [64].

As mentioned above, the development of CDK has been associated with overexposure to UVR [65]. However, association does not necessarily mean causation. To infer causality, it is necessary to conduct field studies to assess the individual dose-response to UVR. The values of UVR can be calculated at ground level using radiometers or computer programs that manipulate different variables such as ozone, latitude, date, time, and cloudiness, among others [66]. The estimation of UVR dose reaching the eyes is also very important in order to be able to determine its harmful effects. However, getting these values requires the installation of equipment with constant evaluation by qualified staffs, which have to simultaneously record climatic factors, such as clouds and winds. Given that CDK is a chronic disease of slow evolution, we should had made all these measurements for many consecutive years in the four extremely isolated Argentinean regions we investigated, which was certaintly an unfeasible task. For these reasons, in the present investigation we did not calculate the UVR values at ground level for any of the four Argentinean regions. Instead, we determined the total O_3_ column concentration for these regions during 10 years using the website of the National Aerospatial Agency (NASA, USA), and all the O_3_ values found during this extended period of time were within the normal range, excluding the possibility of any thinning in the O_3_ layer in these Argentinean regions which could have increased the amount of UVR reaching the ground surface and affect the individuals corneas.

Our findings also allowed reaching conclusions about the role played by labor activity, diet, and the use of appropriate eyes protection on the genesis of CDK. We observed CDK only in people who worked in sheep farming during their entire life in region 3 of Argentina (characterized by a dry, sunny climate, with sandy and arid soil sparsely covered by small shrubs that only allowed the development of highly adapted plant species, which could be exploited by cattle, but was not suitable for growing vegetables or fruits). Surprisingly, we did not find CDK in region 1 or 2 (provinces of Jujuy and Santiago del Estero), which had similar climate and soils and where individuals have similar eating habits and work activity to those of region 3.

The questionnaire about food consumption clearly indicated a dietary deficiency of rich vitamins foods, especially AA, in individuals from regions 1, 2, and 3. Those answers were corroborated by the low sAA concentrations found in blood samples. In contrast, in region 4 there was a greater agricultural activity, mainly fruit cultivation, using Río Negro water for irrigation. Thus, individuals from this region had a much more balanced diet, as their dietary intake includes meat, vegetables, and fruits, with the consequent higher concentrations of sAA. These results are consistent with other investigations which clearly demonstrate the importance of AA in corneal protection against the damaging effects of UVB [59].

Other observations of our work are related to the use of protective eyewear for UVB (winged hats, visors, or sunglasses), commonly used by individuals from regions 1, 2, and 4, where no CDK was found, but not in region 3, where we found a 20% prevalence of this disease. It should also be borne in mind that regions 1, 2, and 3 are characterized by the presence of extensive areas devoid of shadows.

Based upon our results, which clearly demonstrate the existence of CDK only in one out of four Argentinean regions with similar climates, it should be worth considering that a proper name for this corneal pathology should be environmental droplet keratopathy or environmental proteinaceous corneal degeneration, rather than climatic droplet keratopathy because its genesis is not only related to the climate. The prevalence of this disease is high in this area of the Argentinean Patagonia, but many individuals living in this region, in spite of having the same work activity, nutrition, and eye protection, do not suffer CDK. This implies that beside harsh environmental conditions and lack of protection from the detrimental effect of UVR other factors (genetics) could contribute to the onset of this disease.

In addition, when we studied old adult sheep that graze in the same region of Patagonia where we found this human disease (and therefore were exposed to the same environmental conditions as patients with CDK) it was observed that, despite having superficial corneal abrasions, these sheep did not suffer from this corneal degenerative disorder as observed in humans [67] and our unpublished data. This was probably due to the fact that sheep, unlike humans, are able to synthesize AA from the grass they eat.

The results of the pinguecula and pterygium prevalence (ophthalmoheliosis) were as follows: In people inhabiting region 4 there was the lowest number of pinguecula and pterygium cases, whereas the highest prevalence of both diseases was in region 3 (Table 2).

Classically, it has been postulated that pterygium is in part the result of conjunctival inflammatory process secondary to a sustained exposure to UVR. The high prevalence of this disease in region 3 may be related to the fact that these individuals spend long hours each day outdoors with sheep, being consequently exposed to UVR and high winds without any eye protection. This is reinforced by the findings of lower percentages of pterygium in regions 1, 2, and 4, where individuals usually wear hats/goggles for sun protection.

In summary we present new and convincing results that contribute to the increasing knowledge about the genesis of this human corneal degenerative disease which open the prospect for a new therapeutic strategy in the prevention as well as progression of CDK. Since the only treatment in advanced cases is a corneal transplantation, which in different impoverished regions of the world is not an available option, the implementation of simple preventive measures such as proper eyes protection and adequate diet with normal levels of AA would contribute to eradication of this disease.

## 5. Conclusions

CDK was only found in high prevalence in individuals inhabiting one out of four Argentinean regions (region 3: El Cuy department). The feeding pattern of individuals from regions 1, 2, and 3 was characterized by a very low consumption of vegetables and fruit with a high intake of meat. In contrast, individuals from region 4 generally had a balanced diet (meat, vegetables, cereals, and fruits). Low serum AA levels were found in individuals from regions 1, 2, and 3 (0.27 mg/dL ± 0.13, 0.31 mg/dL ± 0.11, and 0.21 mg/dL ± 0.09, resp.), where the intake of fruits and vegetables is low. These values were lower (*p* < 0.001) than those determined for region 4 (0.719 mg/dL ± 0.446). All the individuals work outdoors during the greater part of the day. The primary work activity is cattle breeding, with the exception being region 4, which has fruit collectors. No CDK was found in regions 1 and 2, which have similar climate and soils. Here, individuals have habits and work activities comparable to those of region 3 but use eye protection. There were no cases of this disease either in region 4 where outdoor work also predominates but individuals have a balanced diet without AA deficit and use eye protection. We also found lower percentages of pterygium in regions 1, 2, and 4, where individuals usually wear hats/goggles for sun protection.

CDK was clearly associated with a poor ingestion of fruits and vegetables rich in AA, low levels of AA in sera, and lack of eye protection from UVB, and not merely with the climate. The O_3_ values determined during the last ten years in all the regions were within the normal range excluding the possibility of any thinning in the O_3_ layer in any of these regions.

Based upon our results, it should be worth considering that a proper name for this corneal pathology should be environmental droplet keratopathy or environmental proteinaceous corneal degeneration, rather than climatic droplet keratopathy.

## Figures and Tables

**Table 1:** Demographic data and prevalence of CDK in the four Argentinean regions.

	Region 1 (*n* = 89)	Region 2 (*n* = 134)	Region 3 (*n* = 125)	Region 4 (*n* = 113)
Ages, Mean ± SD	45.38 ± 17.24	55.88 ± 18.34	54.83 ± 13.93	65.16 ± 11.15
(range)	(20–88)	(48–86)	(20–88)	(41–87)
Gender (F/M)	(39/50)	(48/86)	(52/73)	(36/76)
CDK prevalence	0%	0%	20%	0%
*n* (F/M)	0	0	25 (8/17)	0

**Table 2:** Percentages of pterygium and pinguecula in the four regions of Argentina.

	Region 1 (*n* = 89)	Region 2 (*n* = 134)	Region 3 (*n* = 125)	Region 4 (*n* = 113)
Pterygium				
(+/−)	(12/77)^a^	(21/113)	**(21/104)** ^c^	(10/103)
(%)	(13.48)	(15.7%)	**(16.44%) **	(8.8)
Pinguecula				
(+/−)	(54/35)^b^	(47/87)	**(81/44)** ^d^	(15/98)
(%)	(60.67)	(35.1%)	**(64.75%) **	(13.3)

^a^For regions 1, 2, and 3 pterygium prevalence was significantly lower than pinguecula (*p* < 0.0005).

^b^The prevalence of pinguecula among the four regions was significantly different (*p* < 0.0001).

^c^Pterygium prevalence was significantly higher in region 3 versus region 4 (*p* = 0.0688).

^d^Pinguecula prevalence was significantly increased in region 3 versus region 4 (*p* < 0.0001).

**Table 3:** Main work activity, nutrition, and use of eye protection.

	Region 1 (% of individuals)	Region 2 (% of individuals)	Region 3 (% of individuals)	Region 4 (% of individuals)
Source of livelihood	Sheep and camelid farming 72/89 (80.9%)	Goat rearing, deforestation, and charcoal production 104/134 (77.6%)	**Sheep farming and shearing** 117/125 (93.6%)	Fruit pickers 80/113 (70.8%)

Feeding intake	Meat, quinoa, corn, potatoes, and little milk (100%)	Meat, potatoes, zucchini, and few fruits (100%)	**Sheep meat and a little milk** (100%)	Meat, vegetables, cereals, and fruits (100%)

Eye protection	Winged hats 74/89 (83.1%)	Sunglasses 110/134 (82.1%)	**Almost none** 113/125 (90.4%)	Hat and sunglasses 88/113 (77.9%)
